# Factors contributing to a measles outbreak in a hard-to-reach rural village in Xaisomboun Province,  Lao People's Democratic Republic

**DOI:** 10.5365/wpsar.2022.13.3.874

**Published:** 2022-08-03

**Authors:** Vannida Douangboupha, Philippa L. Binns, Bouaphanh Khamphaphongphane, Virasack Som Oulay, Khanxay Sengsaiya, Thounchay Boupphaphanh, Phonepadith Xangsayarath

**Affiliations:** a National Center for Laboratory and Epidemiology, Vientiane Capital, Lao People’s Democratic Republic.; b National Center for Epidemiology and Population Health, Research School of Population Health, ANU College of Health and Medicine, The Australian National University, Canberra, Australia.; c Pediatric Infectious Disease, Mahosot Hospital, Vientiane Capital, Lao People’s Democratic Republic.

## Abstract

**Objective:**

An increase in measles cases was reported in the north-western of the Lao People's Democratic Republic beginning in January 2019, with outbreaks quickly spreading throughout the country. Following identification of two laboratory-confirmed cases in Xaisomboun Province, we conducted an outbreak investigation to identify factors contributing to the measles outbreak in hard-to-reach Village X.

**Methods:**

Active case-finding was undertaken at the provincial hospital and primary health care centre via a retrospective search through admission logbooks and house-to-house surveys in Village X and surrounding villages. Clinical samples were collected from suspected cases, and data were collected using a standard case investigation form. Vaccine coverage data were reviewed.

**Results:**

Of the 40 suspected measles cases with rash onset during 12 February–27 April 2019, 83% (33/40) resided in Village X and 98% (39/40) were of Hmong–Lu Mien ethnicity. Ages ranged from 22 days to 5 years, with 70% (28) aged < 24 months. Almost half of cases aged 9 to < 18 months (5/11) and 67% (8/12) of cases aged ^3^24 months had received a measles-containing vaccine (MCV). Reported MCV coverage in Xaisomboun for children aged < 1 year in 2017–2018 was < 50%. In 55% (22/40) of cases, case notification was delayed by ^3^6 days. The final case classification comprised 10% laboratory-confirmed, 20% clinically compatible, 60% epidemiologically linked and 10% non-cases.

**Discussion:**

This measles outbreak was likely associated with low immunization coverage, compounded by delays in reporting. Effective strategies are needed to address beliefs about and health literacy barriers to immunization and measles awareness. Such strategies may improve MCV coverage and early diagnosis, enabling timely public health interventions and reducing mortality and morbidity.

Measles has been resurgent throughout the World Health Organization’s (WHO’s) Western Pacific Region in recent years. (*1*) In 2019, outbreaks occurred nationwide in the Lao People's Democratic Republic (Lao People's Democratic Republic PDR), with the first laboratory-confirmed case reported in January 2019 in the north-western. Subsequent cases were identified 1 month later in the central region, with cases quickly spreading throughout the country. Xaisomboun was the 11th province to experience a measles outbreak during the first half of 2019.

Xaisomboun is a mountainous province centrally located in Lao People's Democratic Republic PDR (**Fig. 1**). Established in 2013, it is the smallest province in terms of area (8550 km^2^), population (92 682 people) and population density (10.82 people/km^2^). There are three main ethnic groups (43% are Hmong–Lu Mien, 18% are Mon–Khmer and 31% are Lao People's Democratic Republic–Tai in Xaisomboun compared with national proportions of 8%, 21% and 67%, respectively), each with its own unique language and traditional beliefs. (*2*) During 2012–2017, the under5 mortality rate in Xaisomboun was 51/1000 live births compared with 46/1000 nationally. (*3*) The Lao People's Democratic Republic Social Indicator Survey reported that 54.7% of household members surveyed (*n* = 1606) in Xaisomboun were in the two poorest quintiles, had an average of 3.7 persons/sleeping room, and 66.2% and 17.5% had only basic sanitation services and handwashing facilities, respectively. (*3*)

**Figure 1:**
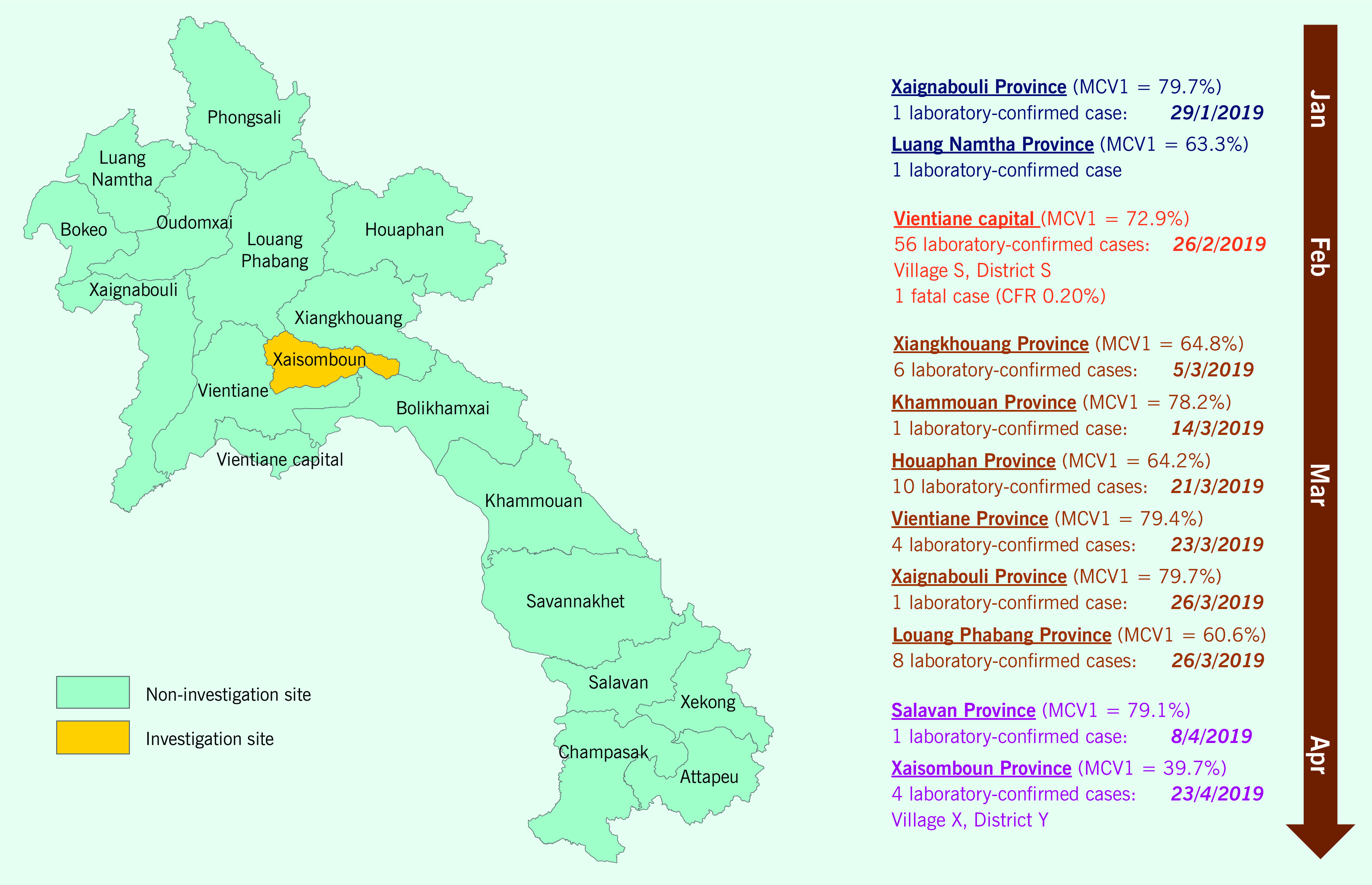
Number of cases of measles and coverage of the first dose of measles-containing vaccine (MCV1), by province, with Xaisomboun Province highlighted, Lao People’s Democratic Republic, 2019

[**insert** Figure 1]

Village X* in Xaisomboun had a population of 2821 people in 2018, 97.1% (*n* = 2739) Hmong–Lu Mien, 2.2% (62) Mon–Khmer and 0.7% (20) Lao People's Democratic Republic–Tai; the population density was 18.2 people/km^2^ and approximately five people slept in one room. (*2*) The village has access to the provincial hospital (a 30-bed secondary care hospital) about 25 km away, and there is one primary health care centre in the middle of the village. (*2*) Traditionally, there is a lot of population movement in and out of the region and between homes during the Hmong–Lu Mien traditional New Year celebrations and for other family events.

Since establishing surveillance for acute fever and rash (AFR) in the province in 2013, no measles outbreaks have been reported. Nine measles/rubella test-negative AFR cases were reported in 2018 and only one in 2019 before the outbreak. (*4*) Additionally, one clinically compatible measles case (test negative) was reported with rash onset on 9 April 2019.

This paper describes the outbreak investigation undertaken after two laboratory-confirmed cases were reported in Village X, Xaisomboun Province, on 23 April 2019.

## Methods

### Definitions

For case-finding, a suspected case was defined as a person who lived in Village X or a nearby area (Villages Y and Z*) who had symptom onset of fever and generalized maculopapular rash between 1 February and 30 April 2019. After suspected cases were identified and investigations completed, standard WHO definitions were applied to determine final case classifications (laboratory-confirmed, epidemiologically linked, clinically compatible and non-measles cases). (*5*) Delayed case notification was defined as ^3^6 days to notification after rash onset. (*6*)

### Case-finding

The 11-person outbreak investigation team included six local public health staff and five central public health and government laboratory staff who travelled from the capital to Xaisomboun by road. The field investigation was carried out from 23 to 30 April 2019. We undertook active case-finding at the provincial hospital and primary health care centre via a retrospective search of admission logbooks and house-to-house surveys in Village X and surrounding villages to identify people fulfilling the definition of a suspected case.

### Data collection and analysis

Face-to-face interviews with caregivers of suspected cases from the three villages were undertaken, guided by the national standard case investigation form. The form is used to collect information about demographics, history of immunization with measles-containing vaccine (MCV), clinical symptoms, complications, hospitalization and treatment outcomes, as well as contacts, travel and participation at gatherings. Vaccination status was identified from the interview or a vaccination card, or both. These descriptive data were analysed using Microsoft Excel (2010).

### Laboratory investigations

Throat swabs and blood samples were collected from suspected cases during their hospital visit or the house-to-house surveys in the three villages. Specimens were transported to the WHO-accredited laboratory at the National Center for Laboratory and Epidemiology (NCLE) and tested for measles and rubella using the Euroimmun anti-measles virus nucleoprotein enzyme-linked immunosorbent assay (ELISA) (immunoglobulin M [IgM]) and the anti-rubella virus glycoprotein ELISA (IgM) (Euroimmun, Lubeck, Germany). Detection of measles virus RNA by reverse transcription–polymerase chain reaction was conducted as described in WHO’s Surveillance standards for vaccine-preventable diseases. (*5*) About 10% of suspected cases were sampled due to the remote location of the village, as per the NCLE’s unpublished standard protocol for measles outbreak investigations.

## Results

### Cases

**Figure 2:**
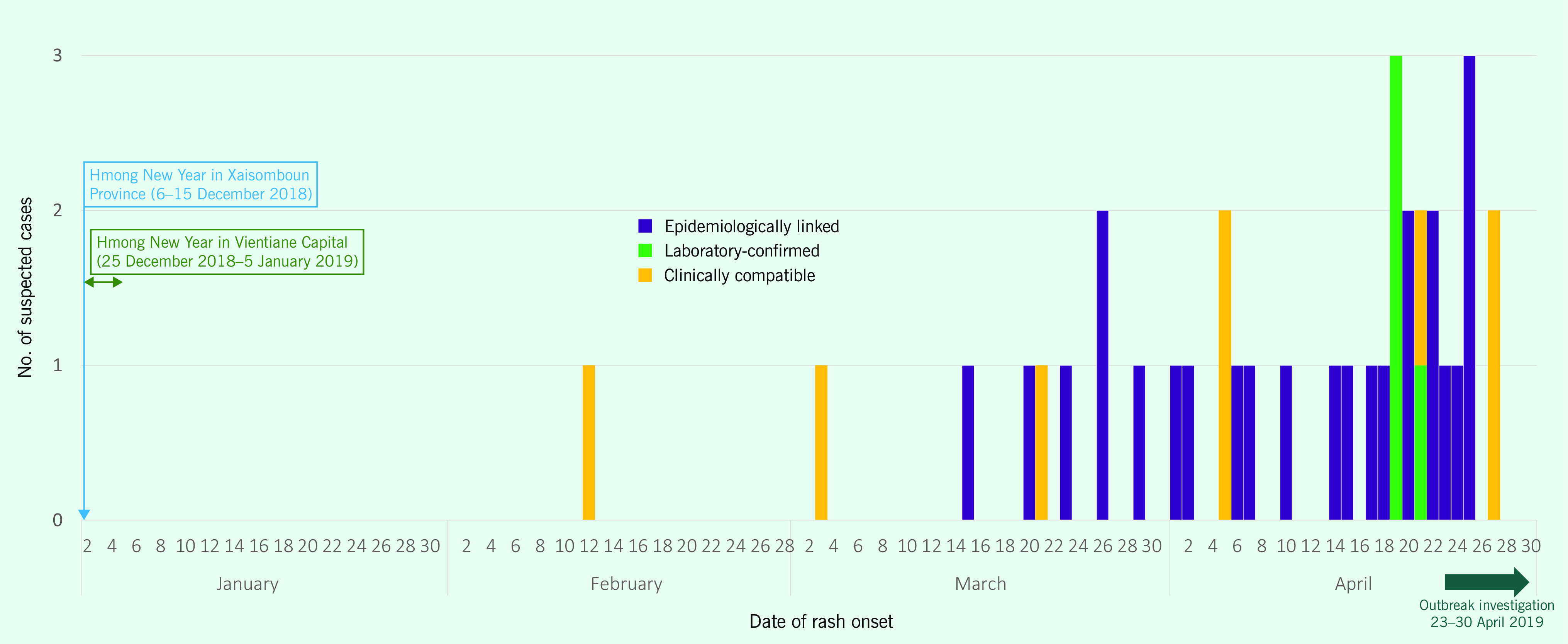
Epidemic curve of the measles outbreak in Xaisomboun Province, Lao People’s Democratic Republic, 1 February–30 April 2019 (N = 40)

Forty suspected measles cases with rash onset between 12 February and 27 April were identified: 10 from the primary health care centre logbook, 24 from the house-to-house survey and six via notifications from hospital clinicians. Four of the 40 initial suspected cases were ultimately classified as non-cases; there were 24 epidemiologically linked cases, eight clinically compatible and four laboratory-confirmed (**Fig. 2**, **Table 1**). The majority of suspected cases resided in Village X (83%; 33) and were of Hmong–Lu Mien ethnicity (98%; 39). All were aged between 22 days and 5 years, with 70% (28) aged < 24 months. There were twice as many males (27) as females (13). The median time from rash onset to notification was 19 days (minimum = 0; maximum = 73; Quartile 1 = 6, Quartile 3 = 31). Altogether, 55% (22) of suspected cases had delayed notification, but all cases had investigation initiated within 48 hours of notification, thus meeting WHO’s surveillance standards. (*5*) There were no reported deaths, but 15% (6/40) of cases were hospitalized at the provincial hospital (**Table 1**).

[**insert** Figure 2]

**Table 1:** Characteristics and risk factors by final case classification for 40 suspected measles cases in Xaisomboun Province, Lao People's Democratic Republic, 1 February–30 April 2019

Characteristic	Laboratory-confirmed^a^	Epidemiologically linked	Clinically compatible	Discarded (non-cases)	Total
Sex					
Female	2 (15.4)	7 (53.8)	2 (15.4)	2 (15.4)	13 (100)
Male	2 (7.4)	17 (63.0)	6 (22.2)	2 (7.4)	27 (100)
Age (months)					
< 9	1 (5.9)	9 (52.9)	5 (29.4)	2 (11.8)	17 (100)
9 to < 18	1 (9.1)	8 (72.7)	1 (9.1)	1 (9.1)	11 (100)
18 to < 24	0	0	0	0	0
^3^24	2 (16.7)	7 (58.3)	2 (16.7)	1 (8.3)	12 (100)
Identification					
Provincial hospital	3 (50.0)	1(16.7)	1 (16.7)	1 (16.7)	6 (100)
Primary health care centre	1 (10.0)	4 (40.0)	4 (40.0)	1 (10.0)	10 (100)
House-to-house survey	0	19 (79.2)	3 (12.5)	2 (8.3)	24 (100)
Immunization status					
Measles-containing vaccine: 1 dose	1 (7.1)	11 (78.6)	1 (7.1)	1 (7.1)	14 (100)
Measles-containing vaccine: 2 doses	1 (100)	0	0	0	1 (100)
None	2 (9.5)	11 (52.4)	5 (23.8)	3 (14.3)	21 (100)
Unknown	0	2 (50.0)	2 (50.0)	0	4 (100)
Ethnicity					
Lao People's Democratic Republic–Tai	0	0	0	1 (100)	1 (100)
Hmong–Lu Mien	4 (10.3)	24 (61.5)	8 (20.5)	3 (7.7)	39 (100)
Mon–Khmer	0	0	0	0	0
Village					
X	4 (12.1)	24 (72.7)	3 (9.1)	2 (6.1)	33 (100)
Y	0	0	5 (58.3)	1 (16.7)	6 (100)
Z	0	0	0	1 (100)	1 (100)
Contact with people with fever and rash 7–21 days before rash onset					
Yes	0	12 (70.6)	2 (11.8)	3 (17.6)	17 (100)
No	2 (11.1)	9 (50.0)	6 (33.3)	1 (5.6)	18 (100)
Unknown	2 (40.0)	3 (60.0)	0	0	5 (100)
Travel during 3 weeks before rash onset					
Yes	0	1 (25.0)	1 (25.0)	2 (50.0)	4 (100)
No	1 (3.0)	23 (69.7)	7 (21.2)	2 (6.0)	33 (100)
Unknown	3 (100)	0	0	0	3 (100)
Participation in social events or gatherings					
Yes	0	3 (50.0)	2 (33.3)	1 (16.7)	6 (100)
No	0	18 (69.2)	6 (23.1)	2 (7.7)	26 (100)
Unknown	4 (50.0)	3 (37.5)	0	1 (12.5)	8 (100)
Time to notification					
£5 days	4 (22.2)	7 (38.9)	3 (16.7)	4 (22.2)	18 (100)
^3^6 days	0	17 (77.3)	5 (22.7)	0	22 (100)
Throat swab collected					
Yes	4 (36.4)	0	3 (27.3)	4 (36.4)	11 (100)
No	0	24 (82.8)	5 (17.2)	0	29 (100)
Serum sample collected					
Yes	4 (50.0)	0	1 (12.5)	3 (37.5)	8 (100)
No	0	24 (75.0)	7 (21.9)	1 (3.1)	32 (100)
Hospitalization					
Yes	3 (50.0)	1 (16.7)	1 (16.7)	1 (16.7)	6 (100)
No	1 (2.9)	23 (67.6)	7 (20.6)	3 (8.8)	34 (100)
Days from rash onset to throat swab					
0	0	0	2 (100)	0	2 (100)
1	1 (50.0)	0	0	1 (50.0)	2 (100)
3	3 (50.0)	0	1 (16.7)	2 (33.3)	6 (100)
4	0	0	0	1 (100)	1 (100)
Days from rash onset to serum collection					
0	0	0	1 (100)	0	1 (100)
1	1 (50.0)	0	0	1 (50.0)	2 (100)
3	3 (75.0)	0	0	1 (25.0)	4 (100)
4	0	0	0	1 (100)	1 (100)

^a^ Values are number (%).

### Laboratory results

Of the 40 cases identified through the investigation, specimens were collected from 28% (11; eight from Village X, two from Village Y and one from Village Z). The time from rash onset to serum sample collection for 8 cases and to throat swab for 11 cases ranged from 0 to 4 days (**Table 1**), meeting criteria for specimen collection adequacy as per WHO’s standards. (*5*) All samples were delivered to the NCLE laboratory within 4 days of collection, and the results were reported within 2 days of specimen receipt, also meeting WHO’s performance indicators. (*5*)

Of the four laboratory-confirmed cases, all resided in Village X, and three swabs from these cases and all their serum specimens were positive for measles. One laboratory-confirmed case had specimens collected 1 day after rash onset, and the other three had specimens collected on day 3 after rash onset (**Table 1**). All 11 specimens tested negative for rubella.

### Vaccination history

Of those aged 9 to < 18 months, 46% (5/11) had received an MCV through routine immunization, as had 67% (8/12) of those aged (*3*)24 months. Only one child aged (*3*)24 months had received the second dose of MCV. Immunization status could not be determined for four cases (**Table 1**).

### Contact, travel and mass gatherings

Altogether, 43% (17) of suspected cases had a history of exposure to a case with fever and rash during the 7–21 days before their rash onset (**Table 1**). Ten percent (4) had travelled during the 3 weeks before rash onset: two had travelled to Xiengkhouang Province, one living in Village X travelled to an unknown village and one living in Village Y visited Village X (**Table 1**). Fifteen percent (6) of cases had participated in social events or gatherings. There was no report of international travel.

### Population risk factors

In Xaisomboun, MCV coverage rates among those aged < 1 year were < 50% in both 2017 and 2018 compared with 80% nationally. (*7*) Surveyed coverage of one dose of MCV in those aged 12–23 months in Xaisomboun in 2017 was 40%, the lowest of all provinces, compared with 66% nationally. (*3*) Vaccination coverage data were not available for minority groups in this province. However, nationally surveyed coverage rates for measles–rubella vaccine in 2017 for children aged 12–23 months varied according to the ethnolinguistic group of the head of household: coverage was highest among families with a Lao People's Democratic Republic–Tai (74%) head of household and lowest among those with a Hmong–Lu Mien (45%) head of household. (*3*)

The traditional Hmong–Lu Mien New Year celebration was during 6–15 December 2018 in Xaisomboun Province and 25 December 2018–5 January 2019 in Vientiane Capital. This event is locally and nationally recognized as a feast that brings together family members who often live in different regions across the country to renew ties and social bonds, and it is also a time to remember ancestors, to pay repect to family spirits, and to reflect on the passing years and prepare for the new year. (*8*)

### Public health interventions

We assisted local teams in notifying health facilities in the area to trigger a risk assessment and immunization response to enhance the current, local surveillance systems for acute fever and rash and clinical case management strategies and to initiate community engagement, awareness and risk communication activities. We provided health education to parents about home care strategies, sanitation, isolation and when to seek medical attention; initiated vitamin A prophylaxis for the cases; and advised health-care workers (HCWs) to undertake regular home visits and to follow up. Subsequently, the Ministry of Health conducted a mass vaccination campaign in the province beginning in early May 2019.

## Discussion

The outbreak investigation identified 40 suspected measles cases, of whom almost all resided in Village X and were of Hmong–Lu Mien ethnicity. All were children, predominantly aged < 24 months. Among those aged 9 to < 18 months, MCV coverage was < 50%. Notification was delayed for most cases. The source of this outbreak was not determined. Travel and gatherings related to the traditional New Year festival may have been contributing factors, although these occurred well before the 7–21-day incubation period for the first suspected case. It is possible that earlier cases related to festival attendance were undetected or, more likely, there was importation from travellers visiting or returning to Xaisomboun from provinces experiencing outbreaks in January and February 2019, such as in Xiengkhouang.

Low MCV coverage likely contributed to the outbreak. We verified that the province had not achieved the recommended MCV coverage of 95%. Contributing factors to low vaccination coverage, all of which are relevant to Xaisomboun, include financial barriers to vaccination, (*9*) being a member of an ethnic minority group with linguistic and cultural barriers, (*10*) lack of knowledge or a low level of education, (*9*) difficulty accessing vaccination centres (*9*) and socioeconomic inequalities. (*11*) Reported factors affecting vaccine compliance in Lao People's Democratic Republic PDR may also relate to vaccine provision and include problems with the supply of vaccines and diluents, the cold chain, lack of availability of HCWs, and capacity issues affecting coordination between relevant organizations to assess needs and make appropriate decisions. (*9*) Vaccine failure (*5*) and vaccine quality are other possible contributing factors. A reduction in immunogenicity related to suboptimal vaccine handling and poor immune response during the national measles campaign in 2011 have previously been proposed and may also be contributory. (*12*)

Additional likely contributing factors to the outbreak include delayed reporting and a lack of case recognition and infection prevention and control measures, as well as hesitancy to seek health care. Delayed reporting as a contributing factor is supported by the number of clinically and epidemiologically linked cases identified during the investigation and the atypical progressive-source epidemiological curve. Two barriers recognized to hinder the provision of high-quality care by HCWs, namely lack of provider education and necessary equipment, may have contributed to the lack of case recognition. (*13*) Inadequate infection prevention and control measures in the health-care setting can contribute to increased measles transmission and spread; however, we did not formally review these measures. Furthermore, poor health-seeking behaviour is associated with low income (*14*) and poor health insurance coverage. (*15*) Evidence indicates that in Lao People's Democratic Republic PDR there is limited coverage of the health equity fund, which creates challenges to accessing health services for those in the poorest quintiles. (*10*) Evidence also suggests that the use of health-care services in Lao People's Democratic Republic PDR often results in financial hardship for patients and their relatives. (*14*) Uninsured people frequently use traditional medicine and self-medication due to perceptions of high prices and poor quality associated with public health services. (*14*) Although there is a relatively robust network of public health services, access can be hindered by the mountainous terrain and lack of year-round roads. (*10*) Overcrowded environments and inadequate sanitation in Xaisomboun, as identified by the Lao People's Democratic Republic Social Indicator Survey, (*3*) also facilitate measles virus transmission. (*16*) The survey (*3*) also suggests that the community in Xaisomboun is hesitant to seek health care, as it reported that for children aged 0–59 months, advice or treatment was not sought for 48.4% of children with diarrhoea and 72% of those with fever compared with national rates of, respectively, 51% and 42%. (*3*) The unique languages and traditional beliefs of the Xaisomboun community may also play an important part in their health-seeking behaviour.

This study highlights issues similar to those identified in other measles outbreaks in the Asia–Pacific region. The 2013–2014 measles outbreak in northern Viet Nam reportedly started among ethnic minorities in mountainous areas that had limited access to vaccination. (*17*) A measles outbreak investigation in a remote area of the Solomon Islands in 2014 suggested that reasons for delayed hospital visits included the long distances between home and hospital, complex sociocultural issues and families first consulting traditional healers. (*18*)

This study had limitations. The retrospective nature of the study, relying on voluntarily self-reported information from participants, means that recall bias and an underestimation of reported cases are likely. Also, additional suspected cases may not have been identified if they were absent from their village during the investigation. We encountered linguistic and cultural barriers and had limited access to professionally trained interpreters; therefore, the accuracy of the data could be affected. However, our results are supported by collaborative evidence and field observations.

This outbreak investigation in a rural, mountainous village in Lao People's Democratic Republic PDR highlights many important considerations. Likely contributors to this outbreak include population movement, low immunization coverage, delayed notification, and a lack of case recognition and health-seeking behaviour, as well as socioeconomic factors. The isolated nature of mountainous communities that have limited access to health care, education and other public services (*19*) increases this population’s susceptibility to outbreaks. Effective strategies are needed to enable local health authorities and communities to work together to understand and address barriers to immunization and to raise awareness about measles. These strategies need to include further exploration of the cultural beliefs, health literacy rates and socioeconomic status specific to the province. Understanding and addressing these issues may help to improve MCV coverage and early diagnosis, enabling timely public health interventions to control outbreaks and reduce mortality and morbidity. Additionally, delivering training modules for HCWs that specifically address measles detection and surveillance with review of feedback from participants and serial nationwide measurements of performance by the Ministry of Health could contribute to increasing the standard of primary health care, thereby improving the health of the population of Lao People's Democratic Republic PDR. (*13*)
